# Shenjinhuoxue Mixture Attenuates Inflammation, Pain, and Cartilage Degeneration by Inhibiting TLR-4 and NF-*κ*B Activation in Rats with Osteoarthritis: A Synergistic Combination of Multitarget Active Phytochemicals

**DOI:** 10.1155/2021/4190098

**Published:** 2021-10-21

**Authors:** Xiaoqin Ma, Chenxia Hao, Zhaokang Zhang, Huiting Jiang, Weixia Zhang, Jingjing Huang, Xiaofei Chen, Wanhua Yang

**Affiliations:** ^1^Department of Pharmacy, Ruijin Hospital, Shanghai Jiao Tong University School of Medicine, Shanghai, China; ^2^Department of Pharmacy, Xi'an Children's Hospital, Xi'an, China; ^3^Department of Pharmacy, Shanghai Children's Medical Center, Shanghai Jiao Tong University School of Medicine, Shanghai, China; ^4^School of Pharmacy, Second Military Medical University, Shanghai, China

## Abstract

Osteoarthritis (OA), a highly prevalent chronic joint disease, involves a complex network of inflammatory mediators that not only triggers pain and cartilage degeneration but also accelerates disease progression. Traditional Chinese medicinal shenjinhuoxue mixture (SHM) shows anti-inflammatory and analgesic effects against OA with remarkable clinical efficacy. This study explored the mechanism underlying anti-OA properties of SHM and evaluated its efficacy and safety via in vivo experiments. Through network pharmacology and published literature, we identified the key active phytochemicals in SHM, including *β*-sitosterol, oleanolic acid, licochalcone A, quercetin, isorhamnetin, kaempferol, morusin, lupeol, and pinocembrin; the pivotal targets of which are TLR-4 and NF-*κ*B, eliciting anti-OA activity. These phytochemicals can enter the active pockets of TLR-4 and NF-*κ*B with docking score ≤ −3.86 kcal/mol, as shown in molecular docking models. By using surface plasmon resonance assay, licochalcone A and oleanolic acid were found to have good TLR-4-binding affinity. In OA rats, oral SHM at mid and high doses (8.72 g/kg and 26.2 g/kg) over 6 weeks significantly alleviated mechanical and thermal hyperalgesia (*P* < 0.0001). Accordingly, the expression of inflammatory mediators (TLR-4, interleukin (IL-) 1 receptor-associated kinase 1 (IRAK1), NF-*κ*B-p65, tumor necrosis factor (TNF-) *α*, IL-6, and IL-1*β*), receptor activator of the NF-*κ*B ligand (RANKL), and transient receptor potential vanilloid 1 (TRPV1) in the synovial and cartilage tissue of OA rats was significantly decreased (*P* < 0.05). Moreover, pathological observation illustrated amelioration of cartilage degeneration and joint injury. In chronic toxicity experiment of rats, SHM at 60 mg/kg demonstrated the safety. SHM had an anti-inflammatory effect through a synergistic combination of active phytochemicals to attenuate pain and cartilage degeneration by inhibiting TLR-4 and NF-*κ*B activation. This study provided the experimental foundation for the development of SHM into a more effective dosage form or new drugs for OA treatment.

## 1. Introduction

Osteoarthritis (OA) is the most prevalent joint disease associated with old age, which is affecting around 240 million people worldwide [1]. In elderly people, OA is a leading cause of disability and significantly increases the social and economic burden [2]. OA is primarily attributed to chronic inflammation, which is responsible for multiple OA phenotypes [3]. In OA, inflammation is triggered and developed by an intrinsic interaction between local tissue damage or metabolic dysfunction products, called damage-associated molecular patterns and the innate immune system via pattern-recognition receptors (PRRs) [4].

As an important PRR, Toll-like receptor (TLR-) 4 is widely expressed in chondrocytes and synovial macrophages. During OA, TLR-4 activation triggers the nuclear factor kappa B (NF-*κ*B) pathway with the secretion of inflammatory cytokines and chemokines such as tumor necrosis factor (TNF-) *α* and interleukin (IL-) 6, resulting in pain and cartilage degradation [5, 6]. Furthermore, TLR-4 can mediate the classically activated type 1 (M1) polarization of macrophages, worsening the inflammatory response in OA [7]. Then, a plethora of inflammatory cytokines, such as TNF-*α*, stimulates the expression of the receptor activator of the NF-*κ*B (RANK) ligand (RANKL) on osteoblasts [8]. Study [9] demonstrated that the RANKL/RANK pathway regulates osteochondral crosstalk, involved in OA progression. TNF-*α* also activates transient receptor potential vanilloid 1 (TRPV1) that is an important sensor on peripheral nerve endings and nonneuronal synoviocytes in the knee joint [10]. TRPV1 functions as a molecular integrator of nociceptive stimuli such as thermal and mechanical stimuli abundant in inflamed joints, thus regulating pain and inflammation [11]. Owing to the complex network of inflammatory mediators and multifactorial etiology in OA, disease-modifying OA drugs (DMOADs) targeting a single inflammatory mediator, such as strontium ranelate, an IL-1*β* inhibitor, demonstrated unsatisfactory efficacy with diverse adverse effects. DMOADs that hit multiple inflammatory targets might have great therapeutic potential [12].

Herbal medicines and herb-derived active phytochemicals have recently been novel approaches with great potential for OA therapy, attributing to their chondroprotective and osteoprotective properties [13–15]. For OA treatment, traditional Chinese medicine (TCM), showing effective efficacy with safety, has been gaining more and more interest and recommended in combination with Western medicine by the pharmacologic guidelines of China [16, 17]. Furthermore, compared to Western medicine, TCM treatment significantly increased the total effective rate and decreased the recurrence rate in OA patients (*P* < 0.00001) [18]. TCM, characterized by “holistic therapy,” has multiple phytochemicals that hit multiple targets involved in inflammation of OA, joint activity function, and bone metabolism [19]. By combining the ingredients relying on the “principal (Jun), minister (Chen), assistant (Zuo), and guide (Shi)”, a compound prescription of TCM can yield a synergistic effect for treating diseases via the action on multiple targets and pathways [20]. Shenjinhuoxue mixture (SHM) is derived from “Wei's traumatology” that is a famous Chinese medical sect and selected as an intangible cultural heritage. SHM has been approved by Shanghai Food and Drug Administration to use in OA patients at Ruijin Hospital for decades and provided a good curative effect with safety.

**Figure 1:**
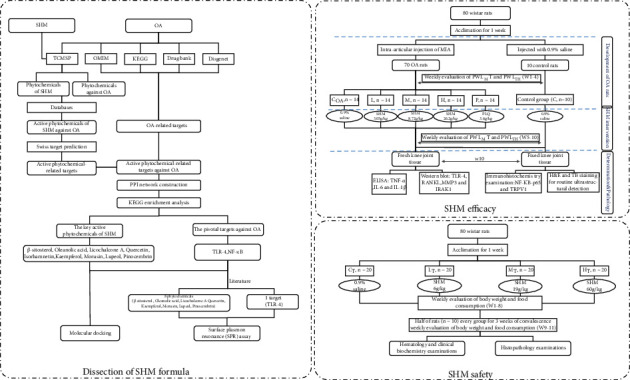
Flowchart of this study.

In a previous study, we predicted that SHM had anti-inflammatory and analgesic actions against OA through multiple active phytochemicals with high oral bioavailability (OB) (OB > 30%) and drug likeness (DL) (DL > 0.18) [21]. These are the criteria for bioactive phytochemicals commonly used in many studies. However, pharmacological activity of some phytochemicals with low OB may be underestimated, such as ursolic acid (OB = 16.77%), because of the enhanced bioactivity through interplays with gut microbiota. In addition, more and more data, including pharmacokinetic (PK) profiles, regarding the anti-OA properties of active phytochemicals in SHM have been reported. Therefore, this study explored further the mechanism underlying synergistic anti-OA properties of SHM and evaluated its pharmacological action and safety in in vivo experiments. The flowchart is shown in Figure 1.

## 2. Materials and Methods

### 2.1. Predicting Mechanism of SHM against OA

#### 2.1.1. Dissection of SHM against OA by Network Pharmacology Analysis and Published Literatures

*(1) Screening of Active Phytochemicals in Herbs of SHM against OA*

The SHM formula is composed by 12 kinds of herbs as follows: *Lycopodii Herba* (*LH*) as the principal; *Radix Angelicae Sinensis* (*RAS*), *Radix Paeoniae Alba* (*RPA*), *Carica papaya* L. (*CPL*), *Frankincense* (*FK*), *Myrrha* (*MH*), and *Radix Gentianae Macrophyllae* (*RGM*) as the minister; *Visci Herba* (*VH*), *Cibot Rhizome* (*CR*), and *Radix Dipsaci* (*RD*) as the assistant; and *Radix Cyathulae* (*RC*) and *Radix Glycyrrhizae* (*RG*) as the guide.

The phytochemicals in SHM herbs active in OA therapy were those present in herbs of SHM as well as those related with OA therapy. First, all the phytochemicals were screened from the traditional Chinese medicine system pharmacology (TCMSP) database (http://lsp.nwu.edu.cn/browse.php) and supplemented by literature from PudMed/MEDLINE, ScienceDirect, Spring Link, Web of Science, CNKI, and VIP database from 2014 to 2020. The active phytochemicals were those with anti-OA activities reported in in vitro or in vivo experiments. High-frequency shared active phytochemicals were identified as the key active phytochemicals based on their possible high levels in SHM.

*(2) Identification of Active Phytochemical-Related Targets of SHM against OA, Herb–Active Phytochemical–Target Network Construction, and Kyoto Encyclopedia of Genes and Genomes Pathway Database Pathway Enrichment Analysis*

The active phytochemical-related targets of SHM against OA were the shared part of the active phytochemical-related targets and the OA-related targets. The targets related to the active phytochemicals in SHM were identified through chemical similarities and pharmacophore models in the SwissTargetPrediction database (http://www.swisstargetprediction.ch). The OA-related targets were collected from the Online Mendelian Inheritance in Man database (http://www.omim.org/,download), the Kyoto Encyclopedia of Genes and Genomes Pathway (KEGG) database http://www.kegg.jp/,download), DisGeNET database (https://www.disgenet.org/search), and DrugBank database (https://www.drugbank.ca/). The protein-protein interactions (PPI) of each active phytochemical-related target against OA were generated using the STRING database. The targets having the interactions with a probabilistic association confidence score of ≥0.9 were selected as the potential targets for further network construction in this study [22]. Network topology parameters (e.g., median and maximum degrees of freedom) in the tab-separated values (TSV) data format using the network analyzer in Cytoscape (version 3.7.1) we used. The key targets with degree values exceeding the mean degree value of the entire network, subjected to KEGG signaling pathway enrichment analysis using the R project, were used. The pathways with *P* of <0.05 were considered statistically significant.

To elucidate the relationship among the herbs, active phytochemicals, and potential targets, the herb-active phytochemical-target network was constructed. Then, these phytochemicals with the highest degree value were also included as the key active phytochemicals. Moreover, the pivotal targets in SHM were those playing a crucial regulatory role in OA treatment.

*(3) Key Active Phytochemical-Target Molecular Docking*

The 2D structures of the key active phytochemicals were downloaded from PubChem database (https://pubchem.ncbi.nlm.nih.gov/). The 2D structure was transferred to a 3D chemical structure to minimize energy for further docking. The 3D structures of the protein targets were downloaded from Protein Data Bank (https://www.rcsb.org/pages/contactus). The proteins were embellished by PyMOL (https://pymol.org/) to remove the original ligand, water molecules, and phosphates. Furthermore, the receptors were prepared by the AutoDockTools (version 1.5.6: http://mgltools.scripps.edu/documentation/links/autodock), including hydrogen addition and docking parameter settings. “Grid box” was set to maximum to perform the blind docking. All ligand and receptor files were saved as pdbqt format.

Finally, AutoDock Vina was used to evaluate and verify the binding affinity of the ligand-receptor relationship. For each ligand, the lowest binding energy was selected as the result of molecular docking. The docking conformation analysis and mapping were performed using PyMOL software, and PLIP (https://projects.biotec.tu-dresden.de/plip-web/plip) was used to analyze the force between ligands and proteins. In general, it is believed that the binding capacity is stronger when the dock binding-free energy is <−4 kcal/mol. The molecular docking model was considered accurate, reliable, and reliable or accurate if its root mean square deviation from the crystal structure was ≤2, ≤4, and<3 Å, respectively [23].

#### 2.1.2. Analysis of Interactions between Active Phytochemicals and Protein Targets by Surface Plasmon Resonance Assay

Surface plasmon resonance (SPR) measurements were performed using a Biacore T200 instrument (GE Healthcare, Uppsala, Sweden). The protein was immobilized on a CM5 sensor chip via the primary amine groups. Before use, the CM5 sensor chip was activated by using sulfo-NHS/EDC chemistry in a buffer (composed of 2.7 mM KCl, 137 mM NaCl, 0.05% (*v*/*v*) surfactant P20, pH 7.4; chemicals and regents were listed in file of *supplementary materials*). The affinity of key phytochemicals to the protein targets was assessed using a Biacore T200 Evaluation Software (version 3.0). The key phytochemicals were flowed at a rate of 30 L/min for 60–180 s to allow for association, followed by another 300 s for dissociation, over immobilized protein in PBS/5% DMSO running buffer (1.05 × PBS, 0.5% P20 surfactant, 5% DMSO, pH 7.4). The phytochemicals were tested for binding at 3.125 *μ*M to 200 *μ*M. Data normalization involved transformation of the *y*-axis such that the theoretical maximum amount of binding for a 1 : 1 interaction with the protein surface corresponded to a sensor response of 100 relative units (RUs).The bound ability of small molecules to the target was evaluated by equilibrium dissociation constant (*K*_D_) [24].

### 2.2. Evaluating the Pharmacological Effects of SHM on the OA Rat Model and Its Potential Chronic Toxicity In Vivo

#### 2.2.1. Chemicals, Reagents, and Animals

SHM was obtained from Ruijin Hospital, Shanghai Jiaotong University School of Medicine (Shanghai, China), and its preparation method with the quality standard was listed in the file of *supplementary materials*. Panlongqi tablets (PLQ) were purchased from Panlong Pharmaceutical Group Limited by Share Ltd. (Shaanxi, China), and monosodium iodoacetate (MIA) was purchased from Macklin Biochemical Technology Co. Ltd. (Shanghai, China). Rat ELISA assay kits of TNF-*α*, IL-6, and IL-1*β* were obtained from Nanjing Jiancheng Bioengineering Institute (Nanjing, China). Anti-TLR-4 rabbit monoclonal antibody, anti-matrix metalloproteinase 3 (anti-MMP3) rabbit monoclonal antibody, and anti-actin mouse monoclonal antibody were bought from Servicebio Technology Company (Wuhan, China). Anti-RANKL rabbit monoclonal antibody was obtained from Boster (Wuhan, China), and anti-IL-1 receptor-associated kinase 1 (IRAK1) rabbit monoclonal antibody was purchased from Proteintech (Wuhan, China). Secondary antibody anti-rabbit HRP, anti-mouse HRP, 5x protein loading buffer, SDS-PAGE gel preparation kit, 5% skim milk, and ECL solution were obtained from Servicebio (Wuhan, China). Reagent absolute ethanol, xylene, hydrochloric acid, ammonia, and neutral gum were obtained from Sinopharm Chemical Reagent Co. Ltd. (Shanghai, China). Hematoxylin-eosin dye (HE) and toluidine blue (TB) were bought from Servicebio (Wuhan, China), and efficient sectioning paraffin was purchased from Shanghai Huayong Olefin Co. Ltd. (Shanghai, China).

In total, 160 healthy-specific pathogen-free Wistar rats aged 8 weeks and weighing 180–200 g were purchased from Xipuer-Beikai Experimental Animal Co. Ltd. (Shanghai, China). All rats were housed in ventilated racks at 24 ± 2°C with relative humidity at 45% ± 5% on a 12 h light/dark cycle (light on from 7 : 00 to 19 : 00). The rats were permitted to acclimate to laboratory conditions at least 1 week before the start of experiments. Animals in pairs were maintained in the cages that were lined with woodchip bedding, and animals were provided with environmental enrichment. Standard solid pellet feed and fresh water were provided ad libitum. All 160 rats were equally divided into pharmacological and chronic toxicity experiments. The experimental protocols were reviewed and approved by the ethics committee of the Shanghai Institute of Pharmaceutical Industry (no. 2019-0051-1 and 2019-0051-2).

#### 2.2.2. Pharmacological Experiment for SHM Effects

Seventy rats were given the intraarticular injection of MIA (for a single dose, 2 mg; dissolved in 50 *μ*L of 0.9% saline) in the right posterior knee to induce the OA model [25]. Then, the knees of rats were bent and straightened alternately for 30 s to disperse MIA throughout the joint. The other 10 rats were intragastrically injected with 0.9% saline as the control (C) group. Subsequently, the mechanical hyperalgesia (MH) and thermal hyperalgesia (TH) of all rats were analyzed weekly to evaluate the rat knee OA models. Rats with significantly lower MH and TH than the C group (*P* < 0.05) for 4 consecutive weeks (W1–4) were considered as osteoarthritic.

All OA rats were randomly divided into five groups on average as follows: (1) OA control (C_OA_), (2) low SHM intervention (L, (3.05 g/kg) 1.4 mL/kg), (3) mid SHM intervention (M, (8.72 g/kg) 4 mL/kg), (4) high SHM intervention (H, (26.2 g/kg) 12 mL/kg), and (5) positive control with (P: PLQ, 3.6 g/kg). SHM, PLQ, and 0.9% saline were orally administered once daily for 6 consecutive weeks (W5–10). The mid-SHM dosage of the rat was approximately 6 times the daily dosage of human [26], and calculations of low-, mid-, and high-SHM dosages of rats were listed in the file of *supplementary materials*. High- and low-SHM dosages were three times and a third of the mid dosage, respectively [27]. In addition, the 10 rats of the C group were administered 0.9% saline orally.

*(1) Analgesia Evaluation by MH and TH Tests*

The MH and TH of OA and C rats were tested weekly during the intervention phase (W5-10). MH and TH were assessed using the electronic Von Frey test (IITC, USA) and thermal plantar tester (IITC, USA), respectively. The electronic Von Frey test was used to automatically record the mechanical force applied to the tip of the device through a manageable force transducer and the paw withdrawal threshold (PWL_MH_) displayed on the screen. The thermal plantar tester automatically deactivated the radiant heat source when the animal withdrew its paw, and the latency was recorded as PWL_TH_. These tests were repeated three times to calculate the average values of PWL in response to the mechanical and thermal forces. In addition, each hind paw was tested with a cutoff time of 20 s during each immersion so as to prevent tissue damage if a rat failed to withdraw its paw.

*(2) Determination of TLR-4, IRAK1, TNF-α, IL-6, IL-1β, RANKL, and MMP3 Levels in Synovial and Cartilage Tissues of OA Rats*

At the end of the experiments (W10), fresh synovial and cartilage tissues were extracted from the posterior knee joints of OA rats and stored at −80°C. All the OA rats were euthanatized before tissue extraction. Then, half of the tissue block of every OA rat was washed with PBS two to three times, cut into small pieces, placed in homogenization tube with one or two small magnetic beads (2 mm), and lysed in 10 times volume of tissue buffer by adding protease inhibitors. The homogenization tube was shaken for 30 min in an ice bath and then centrifuged at 12000 rpm (1 min). Finally, the supernatant, which was the total protein solution, was collected for further analysis.

TNF-*α*, IL-1*β*, and IL-6 levels in the synovial and cartilage tissues of rats were determined using the rat-specific ELISA kits according to the manufacturer's instructions. TLR-4, IRAK1, RANKL, and MMP3 protein levels were measured through Western blot analysis. In brief, total protein was denatured 5 × protein loading buffer in a boiling water bath. Equal amounts of total protein were electrophoresed on a 10% acrylamide SDS gel; they were then electrotransferred onto a PVDF membrane and activated using methanol at a constant voltage of 25 V. After being blocked with skimmed milk powder for 2 h at room temperature, the membranes were incubated at 4°C overnight with antibody specific for the target protein (listed in supplementary materials) and then 1 h with the corresponding secondary antibody (1 : 3000). The membranes were washed three times with TBST and detected with EGL plus kit in a Western blotting detection system. The density of each target band was quantified using Alpha software (Alpha Innotech, USA) and normalized to *β*-actin via optical density.

*(3) Pathological Observation of Synovial Fluid and Cartilage in OA Rats*

The other half of synovial and cartilage tissue was fixed in 25% glutaraldehyde solution for 48 h. Fixed tissues were processed for paraffin embedding, and 3–5 *μ*m thick sections were prepared for immunohistochemical, ultrastructural, and histopathological examination. Immunohistochemistry examination was used to detect NF-*κ*Bp65 and TRPV1 content and distribution. Then, the density of the brown color developed because immunostaining was measured and then statistically analyzed. All sections were examined using a light microscope (Olympus, Tokyo, Japan) with an attached charge-coupled device digital camera (Mingmei Shot 60).

Sample sections were stained with hematoxylin and eosin (H&E) and TB for routine ultrastructural and histopathological examination, respectively, followed by morphometric analysis, at 200x magnification.

#### 2.2.3. Chronic Toxicity Experiment for SHM Safety

Eighty rats were randomly divided into four groups (*n* = 20, 10 males and 10 females) for chronic toxicity analysis as follows: (1) normal control (C_T_), (2) low-dose SHM toxicity (L_T_, 6 g/kg), (3) mid-dose SHM toxicity (M_T_, 19 g/kg), and (4) high-dose SHM toxicity (H_T_, 60 g/kg).

Before administration, SHM was condensed into an extractum (1 g of extractum = 9.6 g of dried medicinal herbs) to achieve a final dilution which was 100 g/1 mL. Here, 0.9% saline and SHM at different doses were given orally to the C_T_ and other groups, respectively, once daily for 8 consecutive weeks.

At the end of the eighth week, half of the rats (*n* = 10, five male and five female) of each group, were euthanatized and whole blood samples were taken from their abdominal aorta for the tests of hematology and clinical biochemistry testing. All organs were weighed before histopathological examinations.

After another 3 weeks, we check the metabolism and elimination of SHM (convalescence). Here, the other 10 rats of each group were maintained and then necropsied after euthanasia for toxicity examination.

*(1) Clinical Observations, Body Weight, and Food Consumption*

All animals in the chronic toxicity experiment were observed twice daily for clinical manifestation of toxicity. In brief, they were checked for changes in the skin, fur, eyes, and mucous membranes. Their mean body weight and food consumption were calculated weekly for each rat individually throughout the testing period.

*(2) Hematology, Blood Coagulation, and Clinical Biochemistry*

Hematologic assessments were performed on an automated hematology analyzer (MEK-6318, Nihon Kohden, Tokyo, Japan), and the results included white blood cell count, red blood cell count, hemoglobin concentration (Hb), hematocrit (HCT), neutrophilic granulocyte percent (NE), lymphocyte percent (LY), monocyte percent (MO), and platelet count (PLT). Serum biochemical parameters, including alanine aminotransferase (ALT), total protein (TP), albumin (ALB), total bilirubin (TBIL), aspartate aminotransferase (AST), total cholesterol (CHO), blood urea nitrogen (BUN), creatinine (Cr), glucose (GLU), sodium (Na), triglycerides (TG), potassium (K), and chloride (Cl), were evaluated using an automated biochemical analyzer (7020, Hitachi, Tokyo, Japan). Prothrombin time (PT) and activated partial thromboplastin time (APTT) for blood coagulation were analyzed on a blood coagulation analyzer (CA-530, Sysmex, Kobe, Japan).

*(3) Histopathology*

The heart, liver, spleen, lungs, kidneys, adrenal glands, brain, thymus, and testes of each rat were weighed. Relative organ weights were calculated as relative organ weight (%) = [organ weight (g)/body weight (g)] × 100.

The peripheral oral cavity, cranial cavity, and all tissues and organs in the thoracic and abdominal cavity were examined visually for any abnormalities. The organs and tissues were fixed in 10% neutral-buffered formalin, embedded in paraffin, sectioned at 3–5 *μ*m thickness, and stained with H&E for microscopic examination. All sections were observed under an optical microscope (Olympus).

### 2.3. Statistical Analysis

All animal data from pharmacologic and toxicity experiments were expressed as means ± SEMs and means ± SDs, respectively. Data of two groups were compared using the parametric Student's *t*-test or the nonparametric Mann–Whitney test. Comparison among three or more groups was performed using one-way analysis of variance, followed by Tukey's post hoc test. All data were analyzed using SPSS for Windows (version 24; IBM, Chicago, IL, USA). The graphs were plotted using GraphPad Prism (version 8; GraphPad, San Diego, CA, USA). *P* < 0.05 was considered statistically significant.

## 3. Results

### 3.1. Anti-Inflammatory Effects of SHM against OA via the Inhibition of TLR-4 and NF-*κ*B Activation

#### 3.1.1. Identification of the Nine Key Active Phytochemicals of SHM and the Key Targets against Inflammation of OA

**Figure 2:**
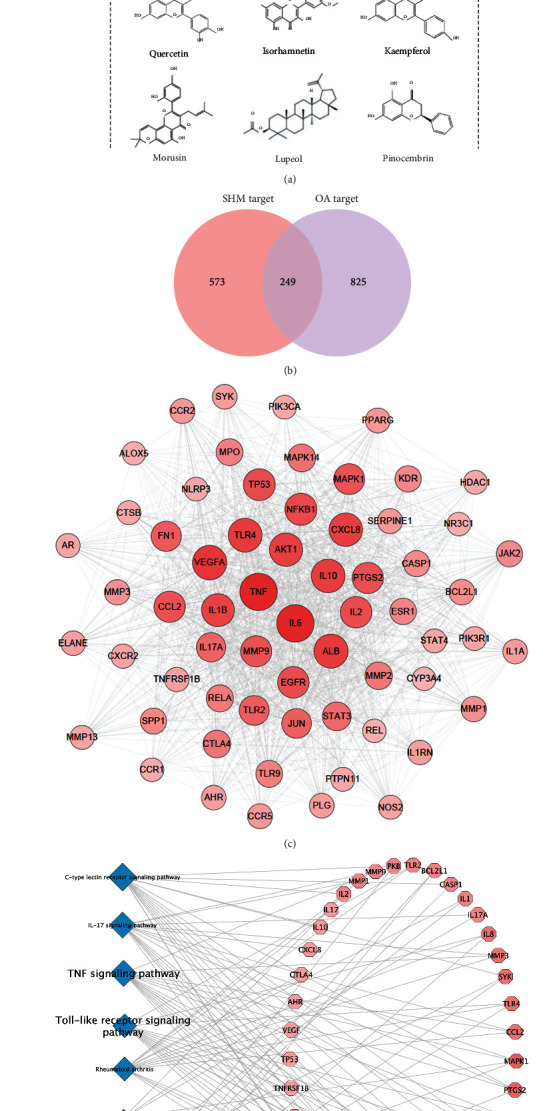
Network pharmacology analysis of the SHM formula. (a) Distribution of active phytochemicals in SHM herbs against OA. (b) The shared targets between SHM potential targets and OA targets. (c) Network of herb-active phytochemical target for SHM against OA. (d) Network of 63 key targets based on central network evaluation. The size of nodes is proportional to the degree centrality by topology analysis. (e) The top ten pathways identified by KEGG enrichment analysis and the corresponding key targets. SHM: shenjinhuoxue mixture; OA: osteoarthritis; KEGG: Kyoto Encyclopedia of Genes and Genomes Pathway database.

In total, 327 phytochemicals in herbs of SHM against OA were obtained from TCMSP database and supplemented by literatures after eliminating the duplicates. There were 67 active phytochemicals, including three supplemented alkaloids (*α*-obscurine, lycojaponicumin C, and lycodoline) of *LH* [28] and one phytochemical (kojic acid) of *CR*. Distribution of active phytochemicals among the herbs of the principal, minister, assistant, and guide in SHM is shown in Figure 2(a). The key active phytochemicals of *β*-sitosterol and oleanolic acid were found in seven herbs (*RAS*, *RPA*, *MH*, *RGM*, *VH*, *RD*, and *RC*) and six herbs (*RPA*, *RGM*, *CPL*, *VH*, *RC*, and *RG*), respectively.

Totally, 249 active phytochemical-related targets against OA were the shared part of 573 active phytochemical-related targets and 825 OA-related targets (Figure 2(b), Supplementary Tables). The PPI network is composed of 173 potential targets and 2699 interactions with an average degree value of 31.2. The 63 key targets (the degree value > 31.2) have 1235 interactions, including the top 10: IL-6, TNF, VEGFA, TLR4, IL-10, CXCL8, AKT1, IL-1*β*, and NF-*κ*B1 (Figure 2(c)). The KEGG analysis results showed that these key targets totally contributed to 98 pathways (*P* value < 0.05).

The top 10 pathways included the TLR signaling pathway, TNF signaling pathway, and NF-*κ*B signaling pathway which were closely correlated with the inflammation and cell death, as shown in Figure 2(d). The herb–active phytochemical–target network (Figure 2(e)) has 254 nodes and 2074 edges. In particular, the key targets of active phytochemicals in assistant and guide herbs included ILs, PIK3R1, and CYP3A4, indicating their anti-inflammation and anti-apoptotic effects and their effects on drug and toxin metabolism.

Based on this network, licochalcone A, quercetin, isorhamnetin, kaempferol, morusin, lupeol, and pinocembrin were also the key active phytochemicals with the highest degree value of 26.9. The results of published studies (Table 1) confirmed that the anti-OA properties of the nine key active phytochemicals involved the inhibition of TLR-4 and NF-*κ*B activation, accompanied by hepatoprotective and renoprotective effects. Thus, TLR-4 and NF-*κ*B were identified as the pivotal anti-OA targets for SHM and they were further analyzed.

#### 3.1.2. Molecular Docking of the Key Active Phytochemicals and the Pivotal Targets of TLR-4 and NF-*κ*B

**Figure 3:**
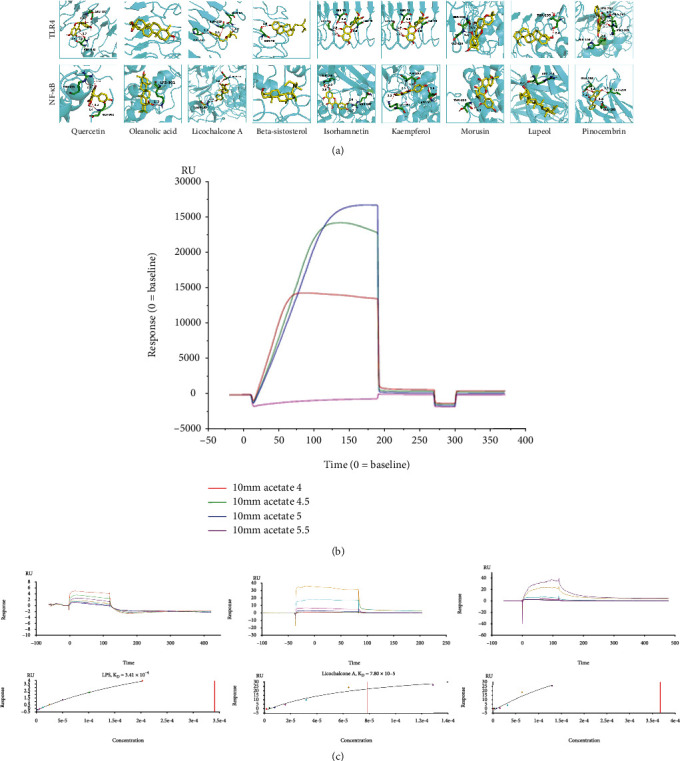
Interactions between the key active phytochemicals and the pivotal targets of TLR-4 and NF-*κ*B. (a) Molecular models of the nine key active phytochemicals (molecule ligands) binding to the proteins of NF-*κ*B and TLR-4. The key active phytochemicals are shown interacting with the 3D structures of proteins, represented by the yellow stick models. Green and blue lines represent residues in the binding sites. The red dashed lines demarcate hydrogen bonds, and the interaction distances are indicated next to the bonds. (b) Sodium acetate pH 5.0 was the optimal condition to dilution TLR-4 with the highest bond response of 3324.5 RU. (c) Affinity-sensing diagrams (on the top) and fitting curves (on the bottom) of a series of concentrations of LPS (positive reference standard), licochalcone A, and oleanolic acid compounds with TLR-4. NF-*κ*B: nuclear factor kappa B; TLR-4: Toll-like receptor 4; LPS: lipopolysaccharide; KD: equilibrium dissociation constant; RU: response units.

All these nine key active phytochemicals could easily enter and bind to the active pocket of the TLR-4 and NF-*κ*B protein as shown in Figure 3(a). The simulated results showed that the most binding complexes were lupeol-TLR-4 docking (−7.03 kcal/mol) and oleanolic acid–NF-*κ*B docking (−8.52 kcal/mol), as listed in Table 2. The hydrogen bond was the main form of interaction. The hydroxyl and carbonyl groups of the phytochemicals formed hydrogen bonds with the proteins.

Moreover, the interaction distances (root mean square deviations) between those phytochemicals and proteins were <3.9 Å, representing the accuracy or reliability of the molecular docking models.

#### 3.1.3. Binding Affinity between TLR-4 and the Phytochemicals of Licochalcone A and Oleanolic Acid

Isorhamnetin of the nine phytochemicals has been proven to block TLR-4 [55]. Thus, the other eight phytochemicals were selected as the candidate phytochemicals for SPR assay to clarify their interactions with TLR-4.

As shown in Figure 3(b), sodium acetate (pH 5.0) was selected as the dilution buffer to dilute TLR-4 to 50 *μ*g/mL in the immobilization assay because it obtained the highest bound response. Moreover, the bound response of the immobilization level was 3324.5 RU. The good affinity between lipopolysaccharide (LPS; positive reference standard) and TLR-4 with a *K*_D_ of 3.41 × 10^−4^ confirmed the activity of TLR-4. The results of the affinity assays showed that the *K*_D_ for the licochalcone A–TLR-4 and oleanolic acid–TLR-4 interaction was 7.80 × 10^−5^ and 3.66 × 10^−4^, respectively, indicating the affinity between these two phytochemicals and TLR-4 (Figure 3(c)).

### 3.2. Pharmacological Effects of SHM against OA and Its Safety in Rats

#### 3.2.1. Anti-Inflammatory and Analgesic Effects of SHM via Inhibiting TLR-4 and NF-*κ*B Activation in OA Rats

*(1) SHM Improved MH and TH of OA Rats*

**Figure 4:**
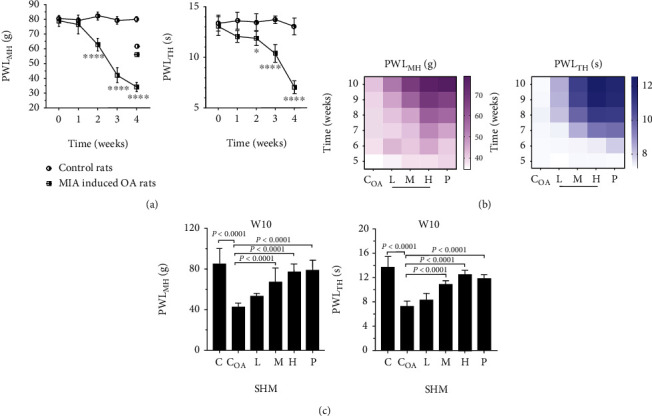
Analysis of mechanical hyperalgesia (MH) and thermal hyperalgesia (TH) in osteoarthritis (OA) rats. (a) Significant declines (*P* < 0.05, weeks 2; *P* < 0.0001, weeks 4) of both paw withdrawal threshold _MH_ (PWL_MH_) and PWL_TH_ in OA rats, compared to control rats, indicated the successful induction of OA by monosodium iodoacetate (MIA). (b) Heat maps of PWL_MH_ and PWL_TH_ in OA rats that received oral shenjinhuoxue mixture (SHM) showed that MH and TH were gradually improved as the dose and treatment course of SHM increased. (c) SHM interventions of mid and high doses significantly increased (*P* < 0.0001) PWL_MH_ and PWL_TH_ in OA rats at the end of pharmacology experiment (weeks 10).

After a single dose of intraarticular injection with MIA, differences in the PWL_MH_ and PWL_TH_ values between the OA and C groups were significant on W2 (*P* < 0.05); the values gradually increased further on W4 (34.2 ± 3.14 g and 7.05 ± 0.631 s for C_OA_ vs. 80.0 ± 1.80 g and 13.0 ± 0.834 s for C, respectively; *P* < 0.0001). These results indicated that our rat OA model was well established (Figure 4(a)).

SHM interventions increased the PWL_MH_ and PWL_TH_ in a time-dose-dependent manner (Figure 4(b)). At the end of the experiments (W10), compared with the C_OA_ group, the groups treated with mid and high doses of SHM showed significant increases in both PWL_MH_ (67.3 ± 13.6 and 76.7 ± 8.43 g, respectively) and PWL_TH_ (10.9 ± 0.245 and 12.6 ± 0.263 s, respectively; all *P* < 0.0001). High-dose SHM showed the similar amelioration of MH and TH with PLQ (P group; PWL_MH_: 78.7 ± 9.98 g, *P* = 0.38 and PWL_TH_: 11.8 ± 0.261 s, *P* = 0.73; Figure 4(c)).

*(2) Effect of SHM on TLR-4, RANKL, IRAK1, TNF-α, IL-6, IL-1β, and MMP3 Levels in the Synovial and Cartilage Tissue of OA Rats*

**Figure 5:**
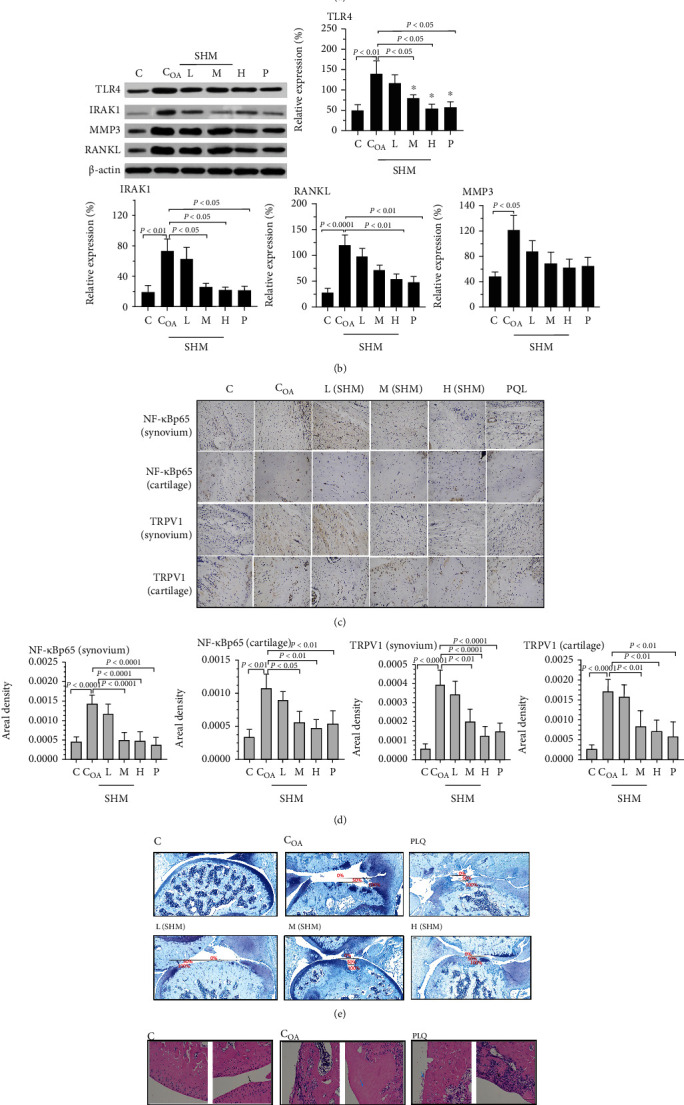
TLR4, RANKL, IRAK1, TNF-*α*, IL-6, IL-1*β*, and MMP3 levels of synovium and cartilage of OA rat knees in C, C_OA_, SHM, and P groups. (a) TNF- *α* , IL-6, and IL-1*β* levels were measured by ELISA kit according to the manufacturer's instructions. (b) TLR4, IRAK1, MMP3, and RANKL levels were measured and quantified by Western blot analysis with *β*-actin as a protein loading control. Pathological changes of synovial and cartilage of rat knees in pharmacology experiment. (c) Representative images of synovium and cartilage sections immunohistochemically stained for nuclear NF-*κ*B-p65 and TRPV1 (dark brown) in OA rats. (d) Quantification of nuclear NF-*κ*B-p65 and TRPV1 levels with their distributions was detected by immunohistochemically staining in synovium and cartilage (*n* = 3). Images of keen joints were stained by (e) TB and (f) H&E.

As shown in Figures 5(a) and 5(b), compared with control rats, OA rats had higher TNF-*α*, IL-1*β*, and IL-6 in the synovial and cartilage tissue (TNF-*α*: 4.28 ± 0.791 ng/L (C) vs. 41.27 ± 6.06 ng/L (C_OA_), *P* < 0.0001; IL-1*β*: 79.0 ± 7.57 ng/L (C) vs. 216 ± 6.38 ng/L (C_OA_), *P* < 0.0001; and IL-6: 52.8 ± 7.44 ng/L (C) vs. 89.0 ± 12.5 ng/L (C_OA_), *P* = 0.002). Notably, SHM interventions at mid and high doses showed the obvious anti-inflammatory effect by reducing the levels of aforementioned cytokines (TNF-*α*: 22.6 ± 7.63 ng/L (mid) and 16.9 ± 5.27 ng/L (high), *P* < 0.01; IL-1*β*: 128 ± 26.8 ng/L (mid) and 96.8 ± 3.71 ng/L (high), *P* < 0.05; and IL-6: 33.8 ± 12.6 ng/L (mid), 28.9 ± 3.50 ng/L (high), *P* < 0.01). The effects were similar to those for PLQ (TNF-*α*, 14.9 ± 5.76 ng/L; IL-1*β*, 100 ± 13.8 ng/L; and IL-6, 37.0 ± 11.0 ng/L) at the end of W10.

In line with these results, the Western blotting results indicated that increased expression of TLR-4, IRAK1, RANKL, and MMP3 was observed in OA rats (*P* < 0.01, *P* < 0.01, *P* < 0.0001, and *P* < 0.05, respectively). Moreover, in the synovial and cartilage tissue, high-dose SHM could significantly inhibit TLR-4, IRAK1, and RANKL expression (*P* < 0.05, *P* < 0.05, and *P* < 0.01, respectively), whereas mid-dose SHM significantly reduced the expression of TLR-4 and RANK (both *P* < 0.05). Additionally, the expression of MMP3 was declined, but nonsignificantly in the SHM and PLQ groups (high vs. C_OA_, *P* = 0.26; P vs. C_OA_, *P* = 0.29).

*(2) Amelioration of Cartilage Injury with the Reduced Expression of NF-κB-p65 and TRPV1 in SHM-Treated OA Rats*

Immunohistochemical staining for nuclear NF-*κ*B–p65 and TRPV1 for detecting their levels and distributions was performed in the synovial and cartilage tissue (Figure 5(c)). NF-*κ*B–p65 and TRPV1 levels were significantly lower in all OA rats treated with high and mid doses of SHM (all *P* < 0.0001; Figure 5(d)). The areas of cartilage injury in different groups were observed through TB staining (Figure 5(e)). The results indicated that SHM ameliorated cartilage degeneration, especially in the mid- and high-dose groups. Finally, the H&E staining results (Figure 5(f)) showed that high and mid doses of SHM remarkably mitigated knee joint injury.

#### 3.2.2. Safety of Long-Term SHM Use in Rats

*(1) No Significant Changes in Clinical Observations, Body Weight, and Food Consumption in SHM-Treated Rats*

Clinical signs of toxicity or mortality were not observed in the SHM and control groups during all 8 weeks. Body weight and body weight gain in both male and female rats in the SHM-treated groups were comparable with those of the control group rats (*P* > 0.05, Figure S1A).

Although the food consumption of male rats in the H_T_ group decreased from W2 to W8, the mean weekly food consumption of male and female rats in the SHM-treated groups was generally comparable with that of rats in the control group (*P* > 0.05, Figure S1B).

*(2) No Abnormalities in Hematological and Biochemical Analyses of SHM Groups*

Hematology of SHM-treated male and female rats was generally comparable with those of rats in the control group (*P* > 0.05). Although few values were significantly different between the groups, they were with their physiological range (Table S1). Similar results were observed for blood coagulation test and biochemical analysis (Table S2).

*(3) Normal Organ Architectures in SHM-Treated Rats*

Relative organ weights demonstrated nearly no differences between male and female rats administered with different SHM doses and C_T_ groups on W8 and W11. Histopathological examinations of the heart, liver, spleen, lungs, and kidneys revealed normal architecture, indicating no morphologic disturbances in SHM-treated rats (Figure S1C).

## 4. Discussion

In our OA rats, 8.72 g/kg and 26.2 g/kg SHM orally significantly improved MH and TH (*P* < 0.0001) and restored joint injury after 6 weeks of treatment. It relieved inflammation in the synovium and cartilage tissue, with the decreased levels of inflammatory mediators, including IRAK1, TNF-*α*, IL-6, and IL-1*β*, via the inhibition of TLR-4 and NF-*κ*B activation. Moreover, the declined expression of RANKL and TRPV1 reduced osteoarthritic osteoblast production and pain. We also identified the key active phytochemicals of SHM, including *β*-sitosterol, oleanolic acid, licochalcone A, quercetin, isorhamnetin, kaempferol, morusin, lupeol, and pinocembrin. Their interactions with TLR-4 and NF-*κ*B were also investigated. In particular, licochalcone A and oleanolic acid were verified to have TLR-4-binding affinity. In the chronic toxicity experiment, SHM at 60 mg/kg remained safe in our rats even after 2 months of intervention.

SHM exerted synergistic anti-inflammatory activity in the treatment of OA through multiple active phytochemicals with multitarget effects on the inhibition of the TLR-4 and NF-*κ*B pathways and the PK behaviors. TLR-4 is the pivotal receptor regulating OA inflammation onset and progress via NF-*κ*B pathway activation [77]. Next, the activated NF-*κ*B is the central contributory factor, accompanied by reactive oxygen species (ROS) generation, in joint inflammation and tissue destruction [78]. TLR-4 can also promote OA progression by activating M1 polarization of synovial macrophages with IL-1*β* and TNF-*ɑ* generation [79]. Notably, our SPR assay showed that licochalcone A and oleanolic acid directly inhibited TLR4 (*K*_D_ = 7.80 × 10^−5^ and 3.66 × 10^−4^), exhibiting the antagonistic effects. In line with this, licochalcone A can inhibit the TLR4/NF-*κ*B pathway and efficiently alleviate inflammatory response in mice with LPS-induced acute liver injury [41]. Oleanolic acid can reduce the levels of TLR-4 and its downstream NF-*κ*B in *Salmonella typhimurium*-induced mouse diarrhea [36]. In addition, lupeol shows good affinity with TLR4 in the molecular docking model. Lupeol downregulated the mRNA and protein expressions of TLR4 with the inhibition of the downstream MyD88 and NF-*κ*B, restraining the release of IL-1*β* and TNF-*ɑ*, in viral myocarditis mice [69]. Isorhamnetin can block the LPS–TLR-4 interaction, alleviate inflammatory responses, and reduce ROS generation in BV2 microglia [55]. Moreover, the other active phytochemicals except morusin inhibited the expression of TLR-4 and elicited their anti-inflammatory activities (Table 1). Of these phytochemicals, quercetin can induce the alternatively activated type 2 (M2) polarization of synovial macrophages, inhibiting inflammation and apoptosis of chondrocytes for cartilage repair after OA [45]. Lu et al. [49] reported that quercetin inhibited the TLR4/MyD88/NF-*κ*B signaling pathway, caused the inhibition of M1 macrophage polarization, and exerted renoprotective effects. Thus, these natural inhibitors and/or potential antagonists of TLR-4 may modulate NF-*κ*B activation and M1/M2 polarizations of macrophages, favoring the use of SHM to treat OA. Most key active phytochemicals were multitarget inhibitors of the NF-*κ*B pathway and exhibited synergistic anti-OA properties. These SHM phytochemicals could suppress NF-*κ*B activation in combination with Nrf2, MAPK, and RANKL, alleviating ROS generation, chondrocyte catabolism, and osteoclast differentiation, respectively. Moreover, Yang et al. [80] recently identified that lycopodium alkaloids, such as lycojaponicumin C, from the principal of *LH* have anti-inflammatory effects, which are positively correlated with the chromatographic peak, through AChE/NF-*κ*B pathway suppression in rats with rheumatoid arthritis. However, the effect of lycopodium alkaloids against OA remains insufficient and warrants further research. The anti-inflammatory activity of SHM also depends on the PK behaviors of active phytochemicals. Lycopodium alkaloids show rapid absorption with a *T*_max_ of 0.79–1.58 h after intragastric administration of *LH* extract in rats [28]. Oleanolic acid, quercetin, isorhamnetin, and lupeol are slowly absorbed into the blood with a long *T*_max_ of 3–7.2 h. Kaempferol has high clearance with *t*_1/2_ of 4.05 ± 0.4048 min and CL of 4.06 ± 0.432 L/h/kg [64]. However, the other phytochemicals are long-acting active phytochemicals with low clearance (e.g., lupeol with *t*_1/2_ = 13.564 ± 2.912 h and CL/F = 29.870 ± 4.596 L/h) [71]. SHM might provide the ongoing anti-inflammatory concentration of active phytochemical for OA therapy, attributing to their difference in PK profiles. Additionally, oleanolic acid, lupeol, and pinocembrin, with high *V*_d_ values of 3371.1 ± 1990.1 L, 595.902 ± 210.773 L, and 478 ± 213 L/kg might be distributed widely, indicating high concentrations in OA rat knees.

Furthermore, active phytochemicals of SHM have altered PK profiles after interactions with gut microbiota, membrane transporters, and metabolizing enzymes and these altered phytochemicals synergistically exert anti-inflammatory activity against OA. The active phytochemicals of SHM with low OB were commonly not absorbed well and interacted with gut microbiota. Although ursolic acid has a poor OB (16.77%), it showed high pharmacological actions, related to its active metabolites transformed by intestinal microbes [81]. Arnoriaga-Rodríguez et al. [82] reported that lupeol (OB = 12.12%) positively regulated the gut bacterial ecosystem via a ClpB-like gene function. Accordingly, inflammation in OA is alleviated through the systemic action of gut microbiota regulated by the phytochemicals [83]. Moreover, the anti-inflammatory activity of SHM may be increased via the effects of active phytochemicals on CYP3A4 and P-gp, which are involved in the modulation of first-pass metabolism and enhancement of oral absorption. CYP3A4 and P-gp are located on the apical membrane of the small intestinal cells [84]. CYP3A4 is a key target in the assistant and guide functions of SHM against OA. It is the most abundant hepatic and intestinal phase I enzyme, which catalyzes the metabolism of a wide variety of endogenous and exogenous compounds [85]. We noted that the enzyme activity of CYP3A4 was inhibited by the key active phytochemicals of SHM. However, glycyrrhizin, the main active phytochemical in *RG* (proportion = 19%), can increase CYP3A4 activity by approximately 30% [86]. Some of the key active phytochemicals, especially those in *RG*, can lower the activity of P-gp, potentially inhibiting efflux transport of potential substrate phytochemicals [87]. P-gp is also expressed in the synovial tissue and can influence the joint disposition of drugs [88]. We also found the regulatory effects of the active SHM phytochemicals on CYP1A, CYP2C, UGT1A, BCRP, MRP2, and so on. Interplays between the active GRR phytochemicals and other ingredients via CYP450s and membrane transporters *in vivo* may uncover the role of the guide on transportation of drug to the desired site in SHM.

Long-term use of SHM is safe even at a high dose. Moreover, SHM has a detoxifying function of acetaminophen (APAP) that is an over-the-counter (OTC) analgesic and recommended by all guidelines as the first line of treatment for people with mild to moderate OA [89]. High-dose SHM at 60 g/kg, based on the effective doses of 8.72 and 26.2 g/kg (in the M and H groups, respectively) remained nontoxic even after 8 weeks of intragastric administration in rats. The key active phytochemicals of SHM itself had no toxicity (*β*-sitosterol, isorhamnetin, kaempferol, and lupeol) [27, 58, 72] or has low toxicity (oleanolic acid, LD50 = 90 mg/kg; licochalcone A, 9 *μ*g/mL (MTT cell viability assays); quercetin, >1500 mg/day with nephrotoxicity). Safety of SHM can somewhat be attributed to its complementary combination of active phytochemicals and targets against OA. The key active phytochemicals of SHM can prevent APAP-induced acute hepatotoxicity or nephrotoxicity by inhibiting the NF-*κ*B and PI3K/Akt pathways or by activating Nrf2 [89–93]. Through the suppression of NF-*κ*B and PI3K/Akt pathways, the antiapoptotic activity of the key active phytochemicals can alleviate hepatic necrosis induced by APAP [90]. Because the activation of the PI3K/Akt pathway enhances the transcriptional activity of NF-*κ*B via the activation of I*κ*B kinase (IKK*α*/*β*) [91]. NF-*κ*B not only participates in the process of immunity and inflammation but also causes apoptosis by regulating apoptotic genes [92]. Furthermore, because of the interactions of ROS with NF-*κ*B and PI3K, the antioxidant activity of these active phytochemicals via the activation of the Nrf2 pathway can relieve inflammation and apoptosis [26, 32, 38, 50, 56, 62, 72, 75, 88]. The pleiotropic activities of the active phytochemicals indicate thus the safety of SHM when it is used alone or in combination with APAP.

This study is limited by the effect of SHM on the expression of osteoprotegerin (OPG), with regard to clarifying OPG's role in the osteochondral crosstalk of OA. As a decoy receptor that binds to RANKL, OPG provides the antiosteoclastogenesis activity. Additionally, the therapeutic effects of SHM against OA are closely related to its active phytochemical contents that may differ in different batches, attributing to the quality difference of herbs in SHM. Although we have identified the key active phytochemicals of SHM and verified their interaction with TLR-4 and NF-*κ*B. Further study is needed to identify the reliable quality markers of SHM from these active phytochemicals for the establishment of the scientific system of SHM quality evaluation [93]. Moreover, future research clarifying the synergistic mechanism of active phytochemicals with the precise dosage in SHM is also warranted.

## 5. Conclusions

**Figure 6:**
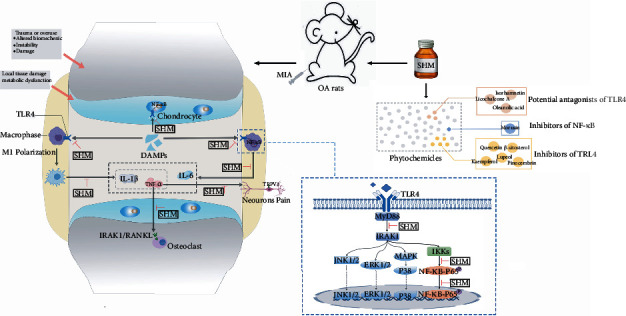
Mechanism underlying treatment of SJHX mixture against OA via inhibiting TLR4 and NF-*κ*B activation.

In summary, our results demonstrated that SHM, derived from the empirical TCM prescription at our hospital, attenuated inflammation, pain, and cartilage degeneration via inhibiting TLR-4 and NF-*κ*B activation in OA rats (the underlying mechanism shown in Figure 6). Moreover, long-term SHM use is safe. The key active phytochemicals in SHM were found to be *β*-sitosterol, oleanolic acid, licochalcone A, quercetin, isorhamnetin, kaempferol, morusin, lupeol, and pinocembrin. Furthermore, licochalcone A and oleanolic acid have antagonistic effect on TLR-4, as verified through their binding affinity. Our study provides an experimental foundation for the development of SHM into a more effective dosage form or DMOADs.

## Figures and Tables

**Table 1:** Pharmacological characteristic of the key active phytochemicals in SHM against OA.

Phytochemicals	Herbs	Molecular targets against OA	Effect on TLR-4	Other pharmacological activities	Pharmacokinetics	OB	DL	Toxicity
*β*-Sitosterol	Minister: *RAS*, *RPA*, *MH*, and *RGM*; assistant: *VH* and *RD*; guide: *RC*	Phosphorylation of NF-*κ*B and the other components of the NF-*κ*B pathway (↓) [29]	Protein expression (↓) [30]	SOD, CAT, and MDA (↓); GSH-P_X_, GSH, and Nrf2 (↑) [31]^a^	*/*	36.91	0.75	No toxicity [32]
Oleanolic acid	Minister: *RPA*, *RGM*, and *CPL*; assistant: *VH*; guide: *RC* and *RG*	SIRT3 (↑) and NF-*κ*B (↓) [33]; MMP-3 (↓); MMP-13, PGE2, IL-6, and caspase 9 (↓), PPARg and SOD2 (↑) [34]; RANKL-induced osteoclastogenesis (↓) [35]	Protein expression (↓) [36]	NF-*κ*B, iNOS, TNF-*α*, IL-1*β*, and IL-6 (↓); MDA (↓); SOD, GSH-px, and Nrf2 (↑) [37]^a^	*C*_max_: 12.12 ± 6.84 ng/mL; *T*_max_: 5.2 ± 2.9 h; *t*_1/2_: 8.73 ± 6.11 h; AUC_0−*t*_: 114.34 ± 74.87 ng/h/mL; CL/F: 555.3 ± 347.7 L/h; *V*_d_/*F*: 3371.1 ± 1990.1 L (dose: 20 mg, oral, human) [38]	29.02	0.76	No influence on C57BL/6 mice at the concentrations below 90 mg/kg [39]
Licochalcone A	Guide: *RG*	Phosphorylation of NF-*κ*B p65 and I*κ*B*α* (↓), iNOS and COX-2 (↓); Wnt/*β*-catenin signaling (↓); Nrf2 and HO-1 (↑); [35, 36] RANKL-induced osteoclastogenesis (↓) [40]	Protein expression (↓) [41]	SIRT1/AMPK (↑) [42], NF-*κ*B, AP-1, and, JNK (↓) [36]; Nrf2 signaling (↑); [41, 43]^a^ CYP3A4, CYP2C9, CYP1A2, CYP2C8, CYP2E1, CYP2D6, and P-gp (↓); BCRP and MRP2 (↑). [41–43]	/	40.79	0.29	No influence on HFF cell viability at the concentrations below 9 *μ*g/mL (MTT cell viability assays) [44]
Quercetin	Minister: *CPL* and *MH*; guide: *RC* and *RG*	M2 polarization of synovial macrophages (↑) [45]; SIRT1/AMPK (↑); SOD and TIMP-1 (↑), MMP-13 (↓) [46]; TLR-4 and NF-*κ*B (↓) [47]; RANKL-induced osteoclastogenesis (↓) [48]	Protein expression (↓) [49]	Akt/NF-*κ*B signaling (↓); PI3K signaling, TLR4/MyD88/PI3K, JAK-STAT, NF-*κB*, p38 MAPK (↓); Nrf2 signaling, and AP-1 (↑) [50]^b^; CYP2E1,CYP3A4,CYP2C19,MRP2,BCRP,P-gp, CYP1A2, and CYP2C8(↓); ABCA1, CYP1A1, and CYP2A6(↑) [51]	*C*_max_: 15.4 ng/ml; *T*_max_: 3 h; *t*_1/2_: 3.47 h; AUC_0−*t*_: 62.5 ng/h/mL; CL/F: 35300 L/h (dose: 500 mg, oral, human) [52]	46.43	0.28	>1500 mg/day with nephrotoxiciy [53]
Isorhamnetin	Assistant: *VH*	ROS production, RANKL-induced osteoclastogenesis, and MAPK/NF-*κ*B/AKT signaling (↓) [54]; NF-*κ*B and p65 (↓) [54]	Inhibiting the bond of LPS with TLR4 [55]	MAPK, NF-*κ*B signaling (↓), PXR (↑); Nrf2 signaling (↑) [56]^b^	*C*_max_: 75.2 ± 6.9 ng/mL; *T*_max_: 7.2 ± 2.3 h; *t*_1/2_, *t*_1/2*α*_: 8.7 ± 4.7 h; *t*_1/2*β*_: 11.2 ± 2.0 h; *t*_1/2ka_: 8.3 ± 3.5 h; AUC_0−*t*_: 1623.4 ± 464.4 ng/h/mL; CL/F: 0.107 ± 0.061 L/h/kg; *V*_d_/*F*: 1.72 ± 1.06 L/kg; *V*_1_/*F*: 1.55 ± 0.48 L/kg (dose:1.00 mg/kg, oral, rats) [57]	49.60	0.31	No toxicity [58]
Kaempferol	Minister: *RPA*; guide: *RG*	NF-*κ*B (↓) [59]; MAPK-associated ERK and P38 signaling (↓) [60]	Protein expression (↓) [61]	TLR4/MyD88/NF-*κ*B P65 signaling (↓); SOD, GPx, GCLC, and Nrf2 signaling (↑); UGT1A1, CYP3A4, P-gp, and CYP2E1 (↓) [62, 63]	*t*_1/2_: 4.05 ± 0.4048 min; AUC_0−*t*_: 992 ± 107 ng/h/mL; CL: 4.06 ± 0.432 L/h/kg; *V*_d_: 0.396 ± 0.0624 L/kg (dose: 4 mg/kg, iv, rats) [64]	41.88	0.24	No data from in vivo studies evidencing these effects [65]
Morusin	Guide: *RG*	NF-*κ*B signaling (↓) [66]	NONE	CYP3A4, CYP1A2, CYP2C9, CYP2E1, UGT1A6, UGT1A7, and UGT1A8 (↓) [67]	/	11.52	0.76	Unknown
Lupeol	Minister: *MH*; assistant: *VH*	RANKL, phosphorylation of MAPK, and NF-*κ*B signaling (↓) [68]	Protein expression (↓) [69]	Phosphorylation of p38 MAPK, JNK, TLR4/MyD88/NF-*κ*B P65 signaling, IRAK (↓), PI3K/Akt signaling, and caspase-3 activity; ROS (↓) and Nrf2 (↑) [70]^b^	*C*_max_: 8.071 ± 2.93 *μ*g/mL; *T*_max_: 6.444 h; *t*_1/2_: 13.564 ± 2.912 h; AUC_0−*t*_: 71.387 ± 7.14 *μ*g/h/mL; CL/F: 29.870 ± 4.596 L/h; *V*_d_/*F*: 595.902 ± 210.773 L; (dose: 200 ng/kg, oral, CD-1 mice) [71]	12.12	0.78	No toxicity [72]
Pinocembrin	Guide: *RG*	NF-*κ*B signaling (↓) [73]	Protein expression (↓) [74]	PI3K/Akt/NF-*κ*B and MAPK signaling (↓), SIRT3 (↑); Erk1/2-Nrf2 (↑), SOD, MDA, and ROS (↓) [75]^a^	*t*_1/2_ : 3.11 ± 1.21 h; AUC_0−*t*_: 518 ± 170 ng/h/mL; CL/F: 110 ± 31.4 L/h; *V*_d_/*F*: 478 ± 213 L/kg (dose: 50 mg/kg, oral, SD rats) [76]	64.72	0.18	Unknown

Herbs of SHM—*CPL*: *Carica papaya L.*; *CR*: *Cibot Rhizome*; *FK*: *Frankincense*; *MH*: *Myrrha*; *RAB*: *Radix Achyranthis Bidentatae*; *RAS*: *Radix Angelicae Sinensis*; *RC*: *Radix Cyathulae*; *RD*: *Radix Dipsaci*; *RG*: *Radix Glycyrrhizae*; *RGM*: *Radix Gentianae Macrophyllae*; *RPA*: *Radix Paeoniae Alba*; *VH*: *Visci Herba*; AP-1: activator protein-1; AMPK: AMP-activated protein kinase; ABCG2: ATP-binding cassette transporter G2 and also known as breast cancer resistance protein (BCRP); Akt: protein kinase B; AUC_0-t_: area under the concentration time curve from zero to time; COX: cyclooxygenase; CAT: catalase; CL: clearance; CL/F: apparent clearance; C_max_: the maximum plasma concentrations; DL: druglikeness; ERK: extracellular-regulated kinase; GSH: glutathione; GSH-Px: glutathione peroxidase; HO-1: hemeoxygenase-1; IL: interleukin; IRAK: interleukin-1 receptor-associated kinase; I*κ*B*α*: nuclear factor of kappa light polypeptide gene enhancer in B-cell inhibitor, alpha; IRF5: interferon regulatory factor 5; JAK-STAT: Janus kinase/signal transduction and activator of transcription; JNK: Jun N-terminal kinase; MAPK: mitogen-activated protein kinase; MDA: malondialdehyde; MMP: matrix metalloproteinase; MRP2: multidrug resistance protein 2; MyD88: myeloid differentiation primary response gene 88; NF-*κ*B: nuclear factor kappa B; Nrf2: nuclear factor- (erythroid derived 2-) like 2; OB: oral bioavailability; PI3K: phosphatidylinositol-3-kinase; PPAR: peroxisome proliferator-activated receptor; P-gp: P-glycoprotein 1; RANKL: receptor activator of the NF-*κ*B ligand; ROS: reactive oxygen species; SIRT 3: sirtuin 3; SOD: superoxide dismutase; TLR4: Toll-like receptor 4; *T*_max_: time taken to reach the *C*_max_; *t*_1/2_: half-life; *t*_1/2*α*_: the distribution half-life; *t*_1/2*β*_: the elimination half-life; *t*_1/2ka_: the absorption half-life; TNF-*α*: tumor necrosis factor-*α*; UGT: UDP-glucuronosyltransferase; *V*_d_: volume of distribution; *V*_d_/*F*: apparent volume of distribution; *V*_1_/*F*: apparent volume of distribution to the central compartment. ^a^Hepatoprotection; ^b^hepatoprotection and renoprotection.

**Table 2:** Molecular docking scores and bonds of the key active phytochemicals against TLR-4 and NF-*κ*B.

Phytochemicals	TLR-4		NF-*κ*B	
	Docking score (kcal/Mol)	Protein residues of hydrogen bond	Docking score (kcal/Mol)	Protein residues of hydrogen bond
Licochalcone A	−3.89	PRO-145, SER-123, GLN-99	−5.5	ASP-92, ASP-291
Quercetin	−5.28	ASN-137, ASN-143	−4.86	GLU-302, RG-275
Isorhamnetin	−5.65	ILE-48, SER-273, GLY-70	−3.86	ILE-196, ARG-267
Kaempferol	−5.67	SER-240, LYS-239	−5.66	LYS-301, ASP-293, ASP-92
Morusin	−6.63	GLY-124, SER-123	−4.76	THR-322
Lupeol	−7.03	THR-235	−8.19	ARG-263
Pinocembrin	−5.84	LUE-204, PRO-202, MET-201, HIS-199	−5.79	GLU-193, LEU-280, GLU-282
Beta-sitosterol	−5.74	SER-73	−5.89	/
Oleanolic acid	−5.28	/	−8.52	LYS-301

## Data Availability

The data used to support the results of this study are included within the article.

## Supplementary Materials

Supplementary Materials (1) Chemicals and regents of surface plasmon resonance (SPR) assay, (2) preparation method of shenjinhuoxue mixture (SHM), and (3) calculation of low, mid, and high SHM dosages of rats was listed in the file of “*Supplementary Materials*.” Table S1: hematologic analysis of male rats (A) and female rats (B) in the chronic toxicity experiment. Table S2: blood coagulation test and clinical biochemistry analysis of male rats (A) and female rats (B) in the chronic toxicity experiment. Figure S1: S1 results of chronic systemic toxicity experiment. (A) Body weight changes and (B) food consumption changes of male and female rats. (C) Histologic observations of the heart, liver, spleen, lung, and kidney in C_T_ and H_T_ groups on week 8 and week 11.
