# Supplementary material for: Shenjinhuoxue Mixture Attenuates Inflammation, Pain, and Cartilage Degeneration by Inhibiting TLR-4 and NF-κB Activation in Rats with Osteoarthritis: A Synergistic Combination of Multitarget Active Phytochemicals
Source: Oxid Med Cell Longev. 2021 Oct 21;2021:4190098. doi: 10.1155/2021/4190098 (PMC8589511; doi:10.1155/2021/4190098)
Supplement: Supplementary Materials — (1) Chemicals and regents of surface plasmon resonance (SPR) assay, (2) preparation method of shenjinhuoxue mixture (SHM), and (3) calculation of low, mid, and high SHM dosages of rats was listed in the file of “Supplementary Materials.” Table S1: hematologic analysis of male rats (A) and female rats (B) in the chronic toxicity experiment. Table S2: blood coagulation test and clinical biochemistry analysis of male rats (A) and female rats (B) in the chronic toxicity experiment. Figure S1: S1 results of chronic systemic toxicity experiment. (A) Body weight changes and (B) food consumption changes of male and female rats. (C) Histologic observations of the heart, liver, spleen, lung, and kidney in CT and HT groups on week 8 and week 11. [file 4190098.f1.docx]

**Table S1 Hematologic analysis of male rats (A) and female rats (B) in the chronic toxicity experiment**

| **A** |  | | | | | | | | | | | | | | | | |
| --- | --- | --- | --- | --- | --- | --- | --- | --- | --- | --- | --- | --- | --- | --- | --- | --- | --- |
|  |  | **RBC (× 10^12^/L)** | | **WBC (× 10^9^/L)** | | **Hb (g/dL)** | | **HCT (%)** | | **LY (%)** | | **MO (%)** | | **PLT (× 10^9^/L)** | | **NE (%)** | |
| **Group** | n | **W8** | **W11** | **W8** | **W11** | **W8** | **W11** | **W8** | **W11** | **W8** | **W11** | **W8** | **W11** | **W8** | **W11** | **W8** | **W11** |
| C_T_ | 5 | 7.37±0.17 | 8.73±0.63 | 7.82±1.67 | 6.27±1.10 | 14.56±0.57 | 15.50±1.15 | 45.40±1.46 | 52.14±3.67 | 72.75±5.04 | 64.43±74.94 | 5.78±1.56 | 8.15±1.63 | 852.0±48.8 | 467±57 | 25.60±5.65 | 26.56±6.30 |
| L_T_ | 5 | 7.41±0.14 | 8.18±0.81 | 6.14±0.85 | 6.49±1.07 | 14.64±0.33 | 14.38±1.08 | 44.10±0.82 | 48.66±3.05 | 70.82±2.94 | 67.09±9.08 | 3.27±0.55^*^ | 5.67±1.60^*^ | 688.6±44.0^**^ | 450±20 | 25.01±2.34 | 26.14±7.50 |
| M_T_ | 5 | 7.72±0.28^*^ | 8.31±0.70 | 8.18±0.83 | 6.31±0.70 | 15.40±0.61 | 15.02±0.41 | 45.22±2.34 | 46.96±1.36 | 74.56±2.16 | 58.98±6.69 | 3.77±0.97^*^ | 3.98±1.80^**^ | 749.6±59.8^*^ | 420±22 | 20.97±1.75 | 35.22±7.89 |
| H_T_ | 5 | 7.40±0.38 | 8.35±0.49 | 7.96±1.39 | 5.01±1.03 | 14.980.20 | 15.52±0.45 | 44.421.63 | 48.86±1.58 | 76.68±4.65 | 64.72±3.71 | 3.52±1.35^*^ | 6.01±2.36 | 740.243.9^**^ | 463±45 | 19.46±4.08 | 28.33±5.06 |
| **B** |  |  |  |  |  |  |  |  |  |  |  |  |  |  |  |  |  |
|  |  | **RBC (× 10^12^/L)** | | **WBC (× 10^9^/L)** | | **Hb (g/dL)** | | **HCT (%)** | | **LY (%)** | | **MO (%)** | | **PLT(× 10^9^/L)** | | **NE (%)** | |
| **Group** | n | **W8** | **W11** | **W8** | **W11** | **W8** | **W11** | **W8** | **W11** | **W8** | **W11** | **W8** | **W11** | **W8** | **W11** | **W8** | **W11** |
| C_T_ | 5 | 6.57±0.35 | 6.96±0.54 | 4.79±0.81 | 4.42±1.31 | 12.42±1.51 | 12.80±0.44 | 41.58±2.70 | 43.16±2.18 | 75.01±4.95 | 74.99±6.55 | 4.70±1.91 | 6.53±1.76 | 763.8±70.0 | 429.0±41.3 | 6.57±0.35 | 18.08±6.08 |
| L_T_ | 5 | 6.64±0.26 | 0.61±0.25 | 4.82±0.95 | 3.65±2.16 | 13.46±0.44 | 13.46±0.76 | 39.28±0.59 | 46.28±2.93 | 75.27±9.16 | 68.05±10.69 | 3.90±0.80 | 3.51±1.54^*^ | 700.4±57.9 | 426.3±21.3 | 6.64±0.26 | 27.79±11.22 |
| M_T_ | 5 | 6.75±0.45 | 6.24±0.31 | 5.26±1.04 | 4.78±0.64 | 13.14±0.45 | 12.13±0.43 | 41.60±2.60 | 49.10±0.90 | 80.77±8.83 | 65.75±23.25 | 3.28±1.10 | 4.40±1.24 | 725±50.5 | 375.5±59.4 | 6.75±0.45 | 29.32±24.27 |
| H_T_ | 5 | 7.04±0.15^*^ | 7.51±0.23 | 5.32±1.71 | 4.03±1.12 | 14.46±0.33 | 14.00±0.3^*^ | 42.92±1.98 | 44.50±2.70 | 80.09±7.72 | 72.97±3.60 | 2.84±0.88 | 4.97±0.76 | 714.2±39.8 | 444.6±22.2 | 7.04±0.15^*^ | 21.49±4.18 |

**Note:** Values expressed as mean ± SDs.

**Abbreviations:** Hb, hemoglobin concentration; HCT, hematocrit; LY, lymphocyte percent; MO, monocyte percent; NE, neutrophilic granulocyte percent; PLT, platelet count; RBC, red blood cell count; WBC, white blood cell count.

**Table S2 Blood coagulation test and clinical biochemistry analysis of male rats (A) and female rats (B) in the chronic toxicity experiment**

| **A** | | | | | | | | | | | | | | |
| --- | --- | --- | --- | --- | --- | --- | --- | --- | --- | --- | --- | --- | --- | --- |
|  | | **C_T_ (n=5)** | | **L_T_ (n=5)** | | | **M_T_ (n=5)** | | | | **H_T_ (n=5)** | | | |
| **Parameters** | **W8** | | **W11** | **W8** | **W11** | | **W8** | | **W11** | | **W8** | | | **W11** |
| **ALT (U/L)** | 59.8±4.5 | | 41.2±4.1 | 53.6±5.0 | 36.2±2.4 | | 55.4±5.2 | | 38.4±4.7 | | 58.4±6.5 | | | 38.6±3.0 |
| **AST (U/L)** | 131.0±20.1 | | 184.0±23.5 | 143.4±24.0 | 182.2±11.8 | | 141.4±14.7 | | 194.6±2.1 | | 125.6±29.0 | | | 172.4±19.4 |
| **TP (g/L)** | 53.6±2.6 | | 52.5±2.5 | 50.7±3.7 | 52.2±2.3 | | 52.5±2.2 | | 51.7±2.6 | | 50.4±2.6 | | | 53.7±2.2 |
| **ALB (g/L)** | 39.4±1.4 | | 40.0±1.7 | 38.7±1.3 | 38.9±1.4 | | 39.9±1.3 | | 40.2±1.2 | | 38.8±0.6 | | | 40.5±1.0 |
| **TBIL (μmol/L)** | 2.4±1.4 | | 2.0±0.5 | 1.3±0.6 | 2.2±0.7 | | 1.4±0.6 | | 1.6±0.5 | | 2.0±0.6 | | | 2.6±0.2 |
| **ALP (U/L)** | 568.4±76.0 | | 329.6±87.0 | 556.4±114.4 | 240.6±47.4 | | 604.8±156.0 | | 281.8±49.7 | | 390.8±92.4 | | | 240.2±48.0 |
| **GLU (mmol/L)** | 8.9±1.0 | | 7.1±0.9 | 8.5±0.8 | 6.5±0.7 | | 8.7±0.8 | | 6.9±0.5 | | 8.0±0.5 | | | 6.9±0.7 |
| **BUN (mmol/L)** | 8.0±0.6 | | 4.4±0.4 | 7.2±0.6 | 4.9±0.6 | | 9.4±1.8 | | 5.2±0.5^*^ | | 7.0±0.8 | | | 5.2±0.5^*^ |
| **Cr (mmol/L)** | 26.8±0.8 | | 31.2±3.3 | 23.0±2.5^*^ | 28.4±1.7 | | 30.6±11.7 | | 24.2±2.0^**^ | | 24.2±1.3^*^ | | | 24.2±2.0^**^ |
| **CHO (mmol/L)** | 1.7±0.4 | | 1.22±0.09 | 1.4±0.1 | 1.27±0.24 | | 1.5±0.3 | | 1.14±0.25 | | 1.3±0.2 | | | 1.35±0.26 |
| **TG (mmol/L)** | 1.1±0.8 | | 0.16±0.07 | 1.0±0.4 | 0.16±0.04 | | 0.8±0.1 | | 0.19±0.04 | | 0.4±0.1 | | | 0.19±0.06 |
| **CK (IU/L)** | 901±336 | | 1546±409 | 951.6±208.8 | 1196±165 | | 1010±265 | | 1439±187 | | 732±254 | | | 866±242 |
| **K (mmol/L)** | 5.2±0.3 | | 5.1±0.2 | 4.9±0.3 | 4.9±0.1 | | 4.7±0.2^*^ | | 5.0±0.3 | | 4.5±0.2^**^ | | | 4.9±0.2 |
| **Na (mmol/L)** | 142.8±1.3 | | 147.1±1.1 | 144.6±1.1 | 148.4±1.1 | | 144.6±0.6 | | 149.0±1.0 | | 146.2±0.8 | | | 148.0±1.2 |
| **Cl (mmol/L)** | 102.8±1.5 | | 106.4±0.9 | 105.8±1.3 | 106.6±1.5 | | 106±0.0 | | 107.8±1.1 | | 107.6±0.6 | | | 107.8±1.3 |
| **PT (s)** | 9.4±0.2 | | 9.2±0.3 | 9.5±0.2 | 9.3±0.3 | | 9.1±0.2 | | 9.3±0.2 | | 9.5±0.1 | | | 9.3±0.1 |
| **APTT (s)** | 16.4±1.2 | | 16.0±1.0 | 15.6±0.4 | 16.2±1.1 | | 14.4±2.0 | | 16.8±0.7 | | 16.6±0.5 | | | 14.9±1.3 |
| **B** | | | | | | | | | | | | | | |
|  | **C_T_ (n=5)** | | | **L_T_ (n=5)** | | | | **M_T_ (n=5)** | | | | **H_T_ (n=5)** | | |
| **Parameters** | **W8** | | **W11** | **W8** | | **W11** | | **W8** | | **W11** | | **W8** | **W11** | |
| **ALT (U/L)** | 45.4±10.5 | | 41.0±10.4 | 47.0±4.1 | | 32.4±4.8 | | 45.2±5.8 | | 58.3±32.1 | | 44.6±3.7 | 26.6±4.0^*^ | |
| **AST (U/L)** | 129.2±27.1 | | 139.6±16.7 | 123.4±7.3 | | 136.2±22.2 | | 122.6±13.6 | | 185.0±85.0 | | 119.0±31.3 | 113.8±23.3 | |
| **TP (g/L)** | 53.6±3.8 | | 56.0±2.3 | 53.9±2.4 | | 53.3±4.1 | | 55.9±2.6 | | 58.5±6.7 | | 57.9±2.6 | 53.2±2.2 | |
| **ALB (g/L)** | 44.3±2.8 | | 44.6±1.4 | 46.2±3.3 | | 42.1±1.8 | | 44.6±2.3 | | 40.8±6.6 | | 45.2±3.1 | 44.0±2.4 | |
| **TBIL (μmol/L)** | 1.4±0.7 | | 2.3±0.8 | 1.5±0.4 | | 1.6±0.5 | | 1.5±0.4 | | 2.2±0.1 | | 1.4±0.6 | 2.5±0.8 | |
| **ALP (U/L)** | 431.4±183.6 | | 238.6±60.1 | 362.6±151.4 | | 281.8±49.7 | | 290.0±49.5 | | 611±601 | | 402.6±95.9 | 237.6±55.7 | |
| **GLU (mmol/L)** | 7.9±0.5 | | 5.3±0.8 | 7.4±0.6 | | 5.8±0.7 | | 7.6±0.7 | | 6.3±0.4 | | 8.7±0.7 | 5.4±0.6 | |
| **BUN (mmol/L)** | 8.6±1.4 | | 9.0±0.8 | 7.9±1.0 | | 4.5±0.5^**^ | | 9.3±1.0 | | 9.4±2.2 | | 8.0±0.8 | 8.9±0.9 | |
| **Cr (mmol/L)** | 30.0±5.2 | | 35.8±4.6 | 26.2±3.0 | | 30.0±1.6^*^ | | 27.0±2.7 | | 32.8±6.3 | | 27.6±2.9 | 31.0±3.5 | |
| **CHO (mmol/L)** | 1.8±0.2 | | 1.71±0.20 | 1.9±0.3 | | 1.93±0.18 | | 1.9±0.3 | | 1.50±0.19 | | 1.7±0.3 | 1.59±0.19 | |
| **TG (mmol/L)** | 0.3±0.1 | | 0.27±0.13 | 0.4±0.1 | | 0.26±0.13 | | 0.5±0.1 | | 0.34±0.06 | | 0.2±0.1 | 0.26±0.08 | |
| **CK (U/L)** | 825.4±164.4 | | 1043±193 | 871.4±118.5 | | 994±168 | | 880.6±209.6 | | 787±268 | | 713.4±336.8 | 685±295 | |
| **K (mmol/L)** | 4.3±0.6 | | 4.7±0.2 | 4.2±0.1 | | 4.4±0.3 | | 4.4±0.3 | | 4.5±0.4 | | 4.4±0.4 | 4.7±0.2 | |
| **Na (mmol/L)** | 144.6±1.1 | | 147.2±2.8 | 146.0±1.0 | | 148.8±1.1 | | 146.5±0.6 | | 147.5±1.0 | | 145.4±1.1 | 147.4±0.5 | |
| **Cl (mmol/L)** | 107.0±0.7 | | 106.4±3.1 | 106.8±1.6 | | 109.4±0.9 | | 107.6±1.3 | | 107.3±2.9 | | 108.6±1.6 | 107.6±0.9 | |
| **PT (s)** | 8.9±0.1 | | 8.9±0.2 | 8.6±0.2 | | 8.9±0.1 | | 8.5±0.3 | | 9.0±0.21 | | 9.0±0.4 | 8.9±0.3 | |
| **APTT (s)** | 13.8±0.8 | | 14.6±1.1 | 15.0±1.1 | | 14.1±0.4 | | 14.4±0.9 | | 14.1±0.9 | | 14.0±0.7 | 13.2±1.0 | |
| **Note:** Values expressed as mean ± SDs. | | | | | | | | | | | | | | |

**Abbreviations:** ALB, albumin; ALP, alkaline phosphatase; ALT, alanine aminotransferase; APTT, activated partial thromboplastin time; AST, aspartate aminotransferase; BUN, blood urea nitrogen; CHO, cholesterol; CK, creatine kinase; Cl, chloride; Cr, creatinine; GLU, glucose; K, potassium; Na, sodium; PT, prothrombin time; TBIL, total bilirubin; TG, triglycerides; TP, total protein.
